# Reaching rural communities through ‘Healthy Entrepreneurs’: a cross-sectional exploration of community health entrepreneurship’s role in sexual and reproductive health

**DOI:** 10.1093/heapol/czz091

**Published:** 2019-09-17

**Authors:** Robert A J Borst, Trynke Hoekstra, Denis Muhangi, Isis Jonker, Maarten Olivier Kok

**Affiliations:** 1 Erasmus School of Health Policy & Management, Health Care Governance, Erasmus University Rotterdam, DR Rotterdam, The Netherlands; 2 Department of Health Sciences, Faculty of Science, Amsterdam Public Health Research Institute, Vrije Universiteit Amsterdam, De Boelelaan 1085, HV, Amsterdam, The Netherlands; 3 Department of Social Work and Social Administration, Makerere University, Kampala, Uganda

**Keywords:** Community health workers, community health entrepreneurship, social franchising, social entrepreneurship, sexual and reproductive health

## Abstract

The purpose of the current study was to explore the association between community health entrepreneurship and the sexual and reproductive health status of rural households in West-Uganda. We collected data using digital surveys in a cluster-randomized cross-sectional cohort study. The sample entailed 1211 household members from 25 randomly selected villages within two subcounties, of a rural West-Ugandan district. The association between five validated sexual and reproductive health outcome indicators and exposure to community health entrepreneurship was assessed using wealth-adjusted mixed-effects logistic regression models. We observed that households living in an area where community health entrepreneurs were active reported more often to use at least one modern contraceptive method [odds ratios (OR): 2.01, 95% CI: 1.30–3.10] had more knowledge of modern contraceptive methods (OR: 7.75, 95% CI: 2.81–21.34), knew more sexually transmitted infections (OR: 1.86, 95% CI: 1.14–3.05), and mentioned more symptoms of sexually transmitted infections (OR: 1.83, 95% CI: 1.18–2.85). The association between exposure to community health entrepreneurship and communities’ comprehensive knowledge of HIV/AIDS was more ambiguous (OR: 1.27, 95% CI: 0.97–1.67). To conclude, households living in areas where community health entrepreneurs were active had higher odds on using modern contraceptives and had more knowledge of modern contraceptive methods, sexually transmitted infections and symptoms of sexually transmitted infections. This study provides the first evidence supporting the role of community health entrepreneurship in providing rural communities with sexual and reproductive health care.

**Key Messages**

- Community health entrepreneurship can strengthen the role of lay health workers in providing sexual and reproductive healthcare.
- Rural communities covered by a community health entrepreneurship model had more knowledge of sexually transmitted infections and modern contraceptive methods than communities covered by regular lay health workers.
- Use of modern contraceptive methods was higher among rural communities where Healthy Entrepreneurs were active compared with communities covered by regular lay health workers.
- Community health entrepreneurship may be a more resilient way of organizing community health systems.

## Introduction

The global development community has set sail to reach universal health coverage by 2030 (United Nations General Assembly, 2015). Yet, recent reports signpost that access to essential medicines and health services is currently far from universal (Wagstaff *et al.*, 2016; Wirtz *et al.*, 2017). This seems to hold especially true for sexual and reproductive health services (Fried *et al.*, 2013; Quick *et al.*, 2014; Snow *et al.*, 2015). Barros *et al.* (2012) show that for a substantial part of the sub-Saharan African population so-called ‘modern contraceptive methods’ are out-of-reach. A suggested solution to increase access to such products and services is the strengthening of primary healthcare systems (World Health Organization, 2008). Such systems, however, rely on a functional force of skilled health workers, which often proves to be a significant hurdle for countries where healthcare resources are scarce in general (Jay *et al.*, 2016).

One way to overturn this ‘human resources for health crisis’ is shifting of some formal primary care tasks to lay workers (World Health Organization, 2006). Lay workers are often volunteer community representatives who have been selected to fulfil a role as “community mouthpiece to fight against inequities(…)” (Lehmann and Sanders, 2007). When lay workers are active in primary care, they are usually referred to as ‘community health workers’. Community health workers are often the households’ first point of interaction with the formal health system. Although the exact activities of community health workers differ per setting, they are deemed to be a crucial source of information on sexual and reproductive health (Lewin *et al.*, 2010).

Despite being a promising solution to the international health workforce crisis, community health worker programmes face vast problems in terms of retention and performance. A review by Lehmann and Sanders (2007) even shows that attrition rates in volunteer community health workers can be as high as 77%. In terms of performance, community health workers differ considerably in the role and responsibilities they are able to fulfil. Previous studies describe that such differences often relate to poor-working conditions, lack of supervision and training, irregular supply chains and insufficient remuneration (Glenton *et al.*, 2013; Woldie *et al.*, 2018). Remuneration, in particular, has been a point of debate in the literature (Pallas *et al.*, 2013).

A more sustainable way of organizing sexual and reproductive health services at the primary level could be social entrepreneurship. During the late 1970s, social entrepreneurship programmes proliferated as a means to stimulate social development through an entrepreneurial approach (Mair and Martí, 2006). Such programmes mainly centred around social marketing, social franchising and micro-entrepreneurship as modes to achieve ‘social goals’, including improved health and well-being (Montagu, 2002; Koehlmoos *et al.*, 2009; Beyeler *et al.*, 2013; Firestone *et al.*, 2017). A quasi-experimental study by Khurram Azmat *et al.* (2013) showed that social franchising combined with vouchers effectively increased contraceptive knowledge and use. A systematic review on (social) franchising for health showed mixed effects but concluded that this approach may increase access to reproductive health services (Nijmeijer *et al.*, 2014). Others have argued that social entrepreneurship may be most effective ‘in reaching and sustaining engagement with individuals who may be impossible to reach using a conventional delivery system’ (Tucker *et al.*, 2012).

There is less research that specifically addresses the impact of micro-entrepreneurship in the field of sexual and reproductive health. Sherman and Hunter (2018) did review micro-entrepreneurship approaches but only looked at indirect economic empowerment in relation to preventing HIV infections. Odek *et al.* (2009) offer a similar analysis and conclude that becoming a micro-entrepreneur may result in reduced risk-taking behaviour in some populations. One randomized controlled trial did report on the direct impact of micro-entrepreneurship as an approach to improving health (Nyqvist *et al.*, 2016). However, this study has not been published in the scientific literature and only focused on the effects of micro-entrepreneurship on child mortality in (semi)urban settings. The effectiveness of micro-entrepreneurship in improving the sexual and reproductive health of rural populations remains to be further scrutinized.

In a pursuit to contribute to the understanding of the potential benefits of micro-entrepreneurship for health, this current study set out to explore how the ‘Healthy Entrepreneurs’ community health entrepreneurship programme in rural Uganda affects communities’ sexual and reproductive health knowledge and their use of modern contraceptive methods. The results of this study aim to add to the wider debate on how to organize primary healthcare delivery for rural populations in such a way that it is both sustainable and equitable.

## Materials and methods

### Community health entrepreneurship

This study focused on one particular micro-entrepreneurship model as intervention: the Healthy Entrepreneurs community health entrepreneurship programme. Healthy Entrepreneurs is a Dutch social enterprise aiming to increase access to essential medicines and health services for rural, underserved, populations in fragile settings. Healthy Entrepreneurs is currently active in five low- and middle-income countries, of which four on the African continent and one in Latin America. Their community health entrepreneurship programme in Uganda was first implemented in February 2015 and is still expanding. Using this model, Healthy Entrepreneurs provides essential medicines and health services through a network of micro-entrepreneurs at the community level. The community health entrepreneurship system in Uganda is governed by the main office and a local office of the social enterprise. The implementation is overseen by the local office in partnership with Ugandan civil society organizations.

In Uganda, the community health entrepreneurs are Village Health Team workers (i.e. community health workers) who volunteered to become community health entrepreneurs. The Village Health Team workers receive their generalist training from the Ministry of Health. As part of this training, they also receive a module on sexual and reproductive health (Uganda Ministry of Health, 2002). Earlier studies show, however, that Village Health Team workers often do not receive full training (O’Donovan *et al.*, 2018) or that the content of the trainings is not necessarily updated (Kimbugwe *et al.*, 2014). In addition, financial constraints, among others, have led the Ugandan Ministry of Health to subcontract trainings to international development organizations. Such organizations may focus their trainings in accordance with their specific mandate (e.g. child health or malaria prevention and treatment), which potentially reduces the generalist capacity of the Village Health Team workers (Turinawe *et al.*, 2015).

When Village Health Team workers volunteer to become a community health entrepreneur, they receive additional trainings. The trainings are organized by Healthy Entrepreneurs and Ugandan civil society organizations. The core of this 5-day training focuses on using entrepreneurial skills to uphold and increase the health of the communities served by the community health entrepreneurs. A key theme of the initial training is sexual and reproductive health and rights. During 2 days of the training, the entrepreneurs receive evidence-based training on adolescent health, youth and relationships, pregnancy and safe delivery, antenatal and postnatal care, referral and HIV testing, STI and HIV prevention and treatment, and gender-based violence. This initial training is followed by monthly refresher courses provided by local Healthy Entrepreneurs co-ordinators. The refresher courses are based on the inputs of the community health entrepreneurs (e.g. issues they experience in their community).

Having been trained, the community health entrepreneurs invest a UGX 185 000 (USD 50) starting fee, receive a basket of supplies worth UGX 370 000 (USD 100) on credit and are given a solar chargeable tablet computer. This tablet computer offers several applications for consultation, business management and education. The tablet contains an application with evidence-based educational videos on health, including sexual and reproductive health and rights. These videos are available in both English and local languages, are made with Ugandan actors and cover all the topics that were part of the training of the entrepreneurs. The application for consultations is based on guidelines from the Ugandan Ministry of Health and community health entrepreneurs are required to structure their activities in accordance with these guidelines.

The inclusion of the Village Health Team workers in the community health entrepreneurship model does not change their position in the formal health system (i.e. they remain Village Health Team workers). The community health entrepreneurship model is meant to overcome known issues of the existing Village Health Teams. As such, community health entrepreneurs have two main responsibilities. The first being that the entrepreneurs actively reach out to their communities and provide costless health education and advice—drawing from their trainings and using instructional videos on their digital tablets. In addition, the community health entrepreneurs run micro-pharmacies where community members can buy essential medicines and health products (e.g. antibiotics, modern contraceptives, analgesics, female hygiene products, soap and vitamins).

The entrepreneurs are encouraged to use part of their collected revenue to redeem their 1-year loan. The social enterprise, in their turn, maintains an end-to-end supply chain of guaranteed quality products and medicines—which can be bought by the entrepreneurs at a local Healthy Entrepreneurs warehouse. The opportunity to replenish stocks at the warehouse is combined with monthly peer-meetings and refresher trainings in which the entrepreneurs are required to partake. At the time of this study, the district of focus had 138 active entrepreneurs.

### Study site

The study site was Kibaale district in West-Uganda. The district headquarters of Kibaale are approximately 219 km (136 mi), by road, west of Kampala, Uganda’s capital. The district is accessible from Kampala by road, about three-quarters of which is bitumen tarmac and the rest is unpaved earth. The district is typically rural with only about 1% of the inhabitants living in urban areas. The majority of the population engages in subsistence smallholder farming. The district is remote and underserved in terms of health and other social amenities. The district population was reported at 785 088 persons in 2014 [Uganda Bureau of Statistics (UBOS), 2017].

### Sample size

Power calculations estimated that a sample of 1250 households divided over 25 villages (i.e. clusters) would yield a power of 0.8 to detect 10% difference on key indicators, assuming ρ  =  0.15, α  =  0.05, β  =  0.2 and a cluster size variation coefficient of 0.3 (Hemming and Girling, 2014).

### Study design and participants

This study applied a cross-sectional cohort design. The cohort included households from randomly selected villages within two subcounties of Kibaale district. In order to select a random sample of clusters from all eligible villages, a sampling frame was established. The sampling frame comprised eight villages in subcounty A and 27 in subcounty B, including the verified number of households per village.

A two-stage cluster sampling technique was used to obtain a sample of 250 households in subcounty A and 1000 households in the larger subcounty B, while allowing for unequal probability on selection. First, stratified probability proportional to size sampling was used to select five villages in subcounty A and 20 villages in subcounty B. Healthy Entrepreneurs was active in 13 of these villages. Second, a sampling sequence was generated to ensure a systematic selection of 50 households per village. Research assistants would gather surveys following this predetermined sequence. Eligible participants were adult household representatives of reproductive age (i.e. age 18–49 for females and 18–54 for males).

### Data collection

Data were collected in November 2017 using a tablet-based community impact survey. The survey included modules on household characteristics, respondent demographics, sexual and reproductive health, and general health beliefs and knowledge. The first three modules were derived from the existing 2011 Uganda Demographic and Health Survey (UDHS) [Uganda Bureau of Statistics (UBOS) and ICF International Inc., 2012], whereas the latter was formatted according to the widely used Knowledge, Attitudes and Practices (KAP) model (Amon *et al.*, 2000). The entire survey was translated from English into the local language Runyoro, and subsequently back-translated to confirm unambiguous and valid construction of the survey in both languages. A pilot study was conducted in an area adjacent to the sample regions to trial the survey’s functioning. After piloting, the survey was openly discussed within the team of research assistants and, as a result of the discussion, minor translation and technical errors were resolved.

The data collection process was facilitated by the Open Data Kit (ODK) software packages. ODK is an open-source platform that facilitates both the collection and aggregation of data. Research assistants used Android-based tablet computers in combination with the ODK Collect application to administer surveys. They visited every sampled household and identified an adult man or woman of reproductive age, invited them to voluntarily participate using a standardized script and obtained informed consent prior to survey administration.

### Outcome variables

Five aggregate sexual and reproductive health outcome variables were constructed. All outcome variables were based on official indicators (United Nations Development Group, 2003; Rutstein and Rojas, 2006) and of binary scale; having either a positive score (coded 1) or a negative score (coded 0). The five outcome measures are discussed in detail below.

#### Knowledge of modern contraceptive methods

Household members were asked to mention all contraceptive methods known to them. The total number of ‘modern methods’ mentioned (i.e. female or male sterilization, intrauterine device, injectable, implant, pill, male or female condom, diaphragm and foam) was used to construct an aggregate variable coded 1 if at least one of the modern methods was known, and coded 0 if none of the modern methods were known.

#### Current use of modern contraception

Respondents were asked to indicate which contraception method they were currently using. The total number of modern methods mentioned was used to construct an aggregate variable coded 1 if at least one of the modern methods was currently in use and coded 0 if none of the modern methods were being used.

#### Comprehensive knowledge of HIV/AIDS

Persons who had comprehensive knowledge of HIV/AIDS correctly identified two methods to prevent transmission of HIV, knew that healthy-looking individuals can transmit HIV and rejected two local misconceptions about the transmission and prevention of HIV.

#### Knowledge of sexually transmitted infections

The survey listed five common sexually transmitted infections. Those who mentioned at least one of these infections had a positive score, whereas those who did not mention one of the common infections had a negative score.

#### Knowledge of symptoms of sexually transmitted infections

Those who could identify at least 1 out of 13 symptoms of sexually transmitted infections were identified as having a positive score. Participants who did not mention 1 of the 13 symptoms were coded as having a negative score.

### Exposure variable

All villages in both subcounties had between one and two active community health workers (i.e. a lay health worker governed by the district health office). Healthy Entrepreneurs had been active in a selection of these villages for over a year—recruiting existing community health workers to become a community health entrepreneur. The villages with trained community health entrepreneurs were marked as exposed, whereas villages without community health entrepreneurs were marked as unexposed. The binary exposure variable thus indicated whether a household was located in a village where community health entrepreneurs were active or in a village where community health workers were active. The activity status of community health entrepreneurs was a priori determined using information retrieved from Healthy Entrepreneurs and the District Health Office. This information was cross-validated in informal interviews with the village chairmen and active community health entrepreneurs to prevent contamination of exposure between villages. Besides, it was ensured that the unexposed clusters were not located near the exposed clusters.

### Confounding variable

Following Twisk (2006), a stepped procedure was used to check for potential confounding. A list of potential confounders was established prior to the analyses. Adjusting the model for more than one likely confounder led to unrealistically wide confidence intervals; potentially as a result of overfitting. Therefore, it was decided to adjust the model for the most prominent confounder—which was households’ wealth level. Household wealth was estimated using the International Wealth Index (IWI) as described by Smits and Steendijk (2015). Calculation of the IWI is based not only on how many basic assets a household possesses but also on the household’s access to public utilities such as electricity and safe water. The continuous variable ranges from 0 through 100, with a value of 0 indicating that the household does not possess any assets, nor does it have access to electricity or clean water. Consequently, an index-score of 100 should reflect maximum attainable wealth.

### Statistical analyses

All statistical analyses were carried out using Stata/SE (version 14.2; Stata Corp, College Station, TX, USA). The computation of descriptive statistics was stratified by exposure status. The percentages of positive scores on the outcome measures were calculated for the exposed and unexposed group separately. Confidence intervals around the percentages were estimated according to the Agresti Coull approach (DasGupta *et al.*, 2001).

Mixed-effects univariate logistic regression models were used to assess the association between the exposure variable and each outcome measure. To adjust for the clustering of households within villages, a two-level structure with a random intercept for villages was used. The marginal likelihoods of the observed data were estimated using the generalized linear latent and mixed models (gllamm) procedure with adaptive Gaussian quadrature numerical integration (Rabe-Hesketh and Skrondal, 2012). Both the crude and adjusted estimated regression parameters are presented as odds ratios (OR) with their corresponding 95% confidence intervals.

## Results

**Figure 1:**
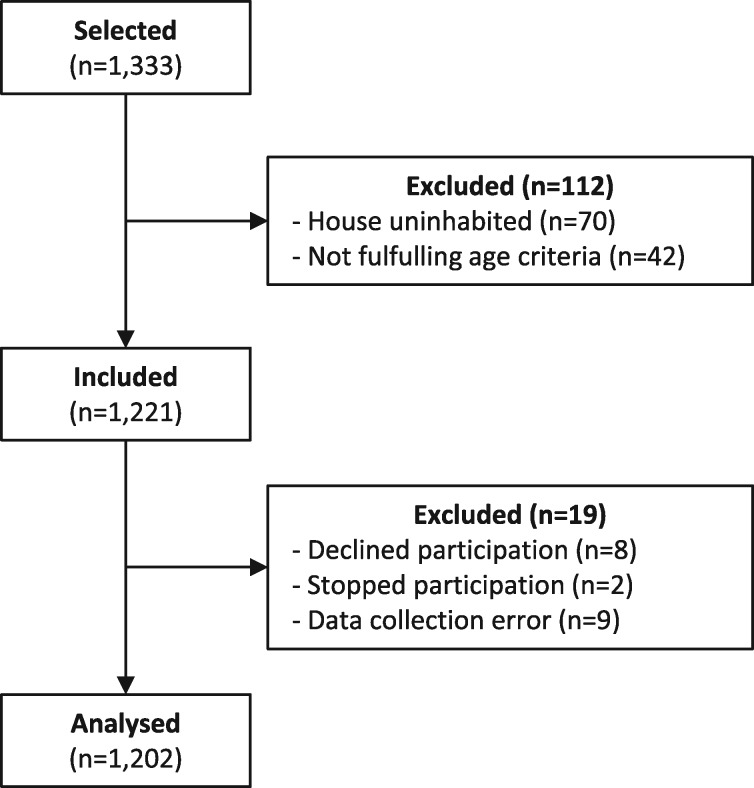
Flow chart of household selection.

The research assistants selected 1333 households for this study. Of this selection, 1221 unique household members were included in the study (see Figure 1). Six participants declined participation for: having no time (*n* = 5) or having no permission from their partner (*n* = 1). Two participants declined participation without providing a reason. Two interviews were ended early because participants had other business to attend to. In nine cases, there were tablet or questionnaire malfunctions. Thus, the final sample contained data from 1202 household members. The response per village ranged from 44 to 57 households. A group of 728 (60%) household members lived in a village with an active community health entrepreneur. The descriptive information of the cohort is presented in Table 1.

**Table 1:** Participant characteristics in the exposed and unexposed clusters

Characteristic	Exposed (*N* = 728)	Unexposed (*N* = 474)
Gender, female, *N* (%)	592 (81.3)	407 (85.9)
Attended school, *N* (%)	631 (86.9)	366 (77.2)
Completed education		
Primary, *N* (%)	184 (25.3)	119 (25.1)
Secondary, or higher, *N* (%)	114 (15.7)	35 (7.38)
Currently married, *N* (%)	289 (39.6)	196 (41.4)
Age in years, males, mean (SD)	30.6 (10.5)	32.0 (11.8)
Age in years, females, mean (SD)	30.7 (10.2)	29.8 (9.2)
IWI, mean (SD)	38.8 (11.9)	32.6 (11.1)

Households living in the villages where community health entrepreneurs were active had higher levels of school attendance (86.9%) and were more often in possession of a diploma from secondary education or higher (15.7%). The exposed and unexposed clusters differed with 6.2 points on the constructed IWI, suggesting that households in the exposed clusters had a higher socioeconomic status. The groups had similar distributions of marital status and age but did differ slightly in gender ratio; with the unexposed group including more women.

**Figure 2:**
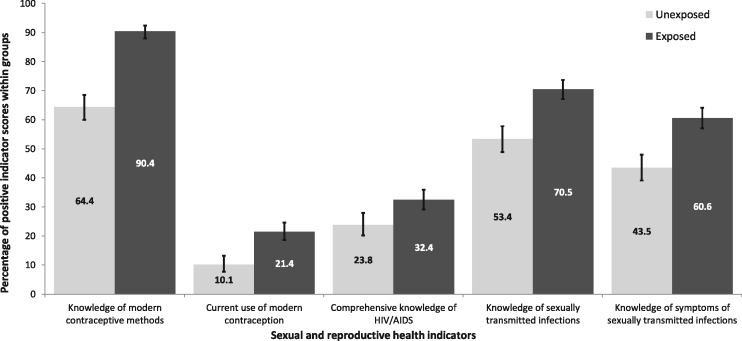
Sexual and reproductive health indicator percentages with 95% confidence intervals per exposure group.

Figure 2 displays the percentage of respondents with positive scores on the sexual and reproductive health indicators, stratified by exposure. The results show that 90.4% (95% CI: 88.0–92.3) of the households living in exposed areas heard of at least one method of modern contraception. Whereas 64.4% (95% CI: 60.0–68.5) of households in the unexposed group was able to mention one method of modern anticonception. In exposed communities, an estimated 21.4% (95% CI: 18.6–24.6) of the households were currently using at least one modern contraceptive method, opposed to 10.1% (95% CI: 7.7–13.2) in the unexposed communities.

Households were, according to the interpretation of the indicators, fairly knowledgeable about sexually transmitted infections and their symptoms. The percentage of household members that was able to mention more than one sexually transmitted infection fluctuated between 53.4% (95% CI: 48.9–57.8) for unexposed and 70.5% (95% CI: 67.1–73.7) for exposed households. The number of participants that knew at least one symptom of sexually transmitted infections was slightly lower with 43.5% (95% CI: 39.1–48.0) in unexposed and 60.6% (95% CI: 57.0–64.1) in exposed communities. A remarkable observation is that both groups seemed to have relatively little comprehensive knowledge of HIV/AIDS with 32.4% (95% CI: 29.1–35.9) and 23.8% (95% CI: 20.2–27.9) in the exposed and unexposed areas, respectively.

The results of the mixed-effects logistic regression analyses on the association between living in areas reached by community health entrepreneurs and sexual and reproductive health are shown in Table 2. Table 2 provides both the crude odds ratios (OR_C_) and the OR adjusted for IWI (OR_A_).

**Table 2:** OR per key survey indicator

Indicator	OR_C_^a^ (95% CI)	*P*	OR_A_^b^ (95% CI)	*P*
1. Knowledge of modern contraceptive methods	11.39 (3.55–36.58)	0.001	7.75 (2.81–21.34)	0.001
2. Current use of modern contraceptive methods	2.41 (1.48–3.92)	0.001	2.01 (1.30–3.10)	0.002
3. Comprehensive knowledge of HIV/AIDS	1.54 (1.14–2.09)	0.005	1.27 (0.97–1.67)	0.09
4. Knowledge of sexually transmitted infections	2.26 (1.27–4.02)	0.006	1.86 (1.14–3.05)	0.013
5. Knowledge of symptoms of sexually transmitted infections	2.13 (1.28–3.53)	0.003	1.83 (1.18–2.85)	0.007

a Crude, for exposed: unexposed.

b Adjusted for IWI, for exposed: unexposed.

The data indicate that the odds on knowing at least one modern contraceptive method were higher for households in the exposed than in the unexposed villages. In addition, a strong association was observed between living in villages exposed to community health entrepreneurs and households’ current use of modern contraceptive methods—even after adjusting for IWI. The results for knowledge on sexually transmitted infections and their symptoms were comparable, though the adjusted ORs are not as extreme. After adjusting for IWI, the between-group difference in a comprehensive knowledge of HIV/AIDS was less univocal.

## Discussion

This study set out to explore how community health entrepreneurship affects communities’ sexual and reproductive health in rural Uganda. The analyses showed that community health entrepreneurship seemed to have a beneficial effect on rural communities’ sexual and reproductive health status. The most salient finding was the association between living in an area where community health entrepreneurs were active and the current use of at least one modern contraceptive method. The proportion of household members reporting to use at least one modern contraceptive method in these areas was over twice as high compared with that of the unexposed areas. Noticeable is that in all cases adjustment for households’ wealth resulted in a decrease of the OR towards nought—suggesting at least some confounding by households’ wealth index.

The healthy entrepreneurs programme is constantly adapting to its environment and this study is limited to a cross-sectional quantitative assessment. Yet, this study is—to the knowledge of the authors—the first rigorous exploration of the role of community health entrepreneurship in providing access to sexual and reproductive healthcare. The observed results suggest that community health entrepreneurship, by providing both sexual reproductive health services and products, does positively affect the sexual and reproductive health status of communities. Comparable to the results from a quasi-experimental study by Hennink and Clements (2005), we observed that the exposed communities had higher scores on the knowledge indicators. Such a difference might be the result of using ‘mobile technology’ (e.g. educational videos on digital tablets) as means of sexual and reproductive health education and sensitization (Braun *et al.*, 2013). An unanticipated finding is that communities have relatively little comprehensive knowledge of HIV/AIDS, especially compared with their knowledge of sexually transmitted infections in general. Although this indicator is based on validated questions, it may be that especially the more tacit knowledge of the household members is not sufficiently captured by these questions (Collins, 2001).

The literature offers little comparable evidence on the wider dynamics of micro-entrepreneurship’s impact on sexual and reproductive health. Most of the evaluated programmes focused solely on micro-financing, not so much on the integrated provision of health services and products through small enterprises (Torri, 2014; Seeley, 2015). Yet, the findings of this study do corroborate observations made in studies that focused on social franchising. Decker and Montagu (2007) showed that such programmes are highly effective in delivering reproductive health services, especially for adolescents. Besides, clinical social franchising and social franchising systems using vouchers have also shown to be successful ways to deliver sexual and reproductive health services and products (Beyeler *et al.*, 2013; Khurram Azmat *et al.*, 2013).

### Reflection on the study

It is important to bear in mind that this study’s cross-sectional design cannot temporally distinguish between exposure and outcome (Flanders *et al.*, 1992). Given that the programme was implemented 1 year prior to data collection, and the programme focused specifically on under-reached villages with a high unmet need, the observed associations are more likely to be an under- than overestimation. Another limitation is potential misclassification in the exposure status. Although exposure status of villages was checked prior to sampling, the non-experimental design of the study does not avert potential contamination in exposure. Although we have no indication that research assistants may have been aware of the villages’ exposure status, this can theoretically induce contamination between intervention and control with subsequent overestimation of the effects.

An important challenge in this study was the chosen regression technique and specifically the potential ‘overfitting’ of the models. Logistic regression models, and generalized linear models with random effects in particular, are susceptible to ‘sparse data bias’ caused by an insufficient number of observations per estimated parameter (Snijders and Bosker, 2012; Greenland *et al.*, 2016). Sparse data bias may have resulted in unrealistically wide confidence intervals surrounding the parameters presented in this study. The potential overfitting proved to be a limitation in including covariates in the primary regression model.

While respecting these limitations, this is the first rigorous exploration of an application of community health entrepreneurship in a rural setting. The likelihood of biased estimators was reduced by using existing survey modules that have been validated in the specific context [Amon *et al.*, 2000; Uganda Bureau of Statistics (UBOS) and ICF International Inc., 2012]. Besides, participants were a random selection of the rural target population and equal chance on selection was ensured.

### Implications and suggestions for further research

The combination of findings provides the first supporting evidence for community health entrepreneurship as an approach to provide rural communities with essential medicines and sexual and reproductive health services. One issue that emerged from the data, in particular, was the programme’s ability to act as vehicle for education and information on sexual and reproductive health. Previous research has shown that national community health worker strategies often fall short on guiding their community health workers in this role as ‘knowledge agent’ (Standing and Chowdhury, 2008). This highlights the potential for community health entrepreneurship as an approach to overcome this challenge and act as a mediator between available knowledge and the communities who could benefit from this knowledge.

As with all efforts to capture the ‘impact’ of interventions in complex and networked health systems, exploring the direct association between community health entrepreneurship and its outcomes remains a challenge. With this study, a robust steppingstone is provided for future longitudinal and experimental research into the outcomes of community health entrepreneurship. Experimental designs, in particular, may reduce the likelihood on confounding by means of randomization. In addition, more in-depth—particularly qualitative—research is needed into the interactions between community health entrepreneurship approaches and existing (public) initiatives for delivery of primary care (Beyeler *et al.*, 2013; Musinguzi *et al.*, 2017). This includes studying more tacit knowledge through observations. In addition, evidence indicates that communities are willing to pay for health products and services (Turinawe *et al.*, 2016) but the measurable equitableness of payment schemes in community health worker approaches remains disputed (Shah *et al.*, 2011; Haemmerli *et al.*, 2018; Mumtaz, 2018). It would be worthwhile to explore this aspect further through both quantitative and qualitative inquiry.

## Conclusion

This cross-sectional study explored how community health entrepreneurship affects the sexual and reproductive health status of rural communities. The results showed that communities covered by community health entrepreneurs had higher odds on using modern contraceptives and substantially higher odds on having knowledge of modern contraceptive methods, sexually transmitted infections and symptoms of sexually transmitted infections. This study provides the first evidence supporting the role of community health entrepreneurship in providing rural communities with sexual and reproductive healthcare.

*Ethical approval.* Ethical clearance was obtained from the Mildmay Uganda Research Ethics Committee and Uganda National Council for Science and Technology. In addition, all activities were agreed upon by the District Health Office. In accordance with Dutch and Ugandan law, no further ethical procedures were required.
